# Nonclinical Human Cardiac New Approach Methodologies (NAMs) Predict Vanoxerine-Induced Proarrhythmic Potential

**DOI:** 10.3390/jcdd12080285

**Published:** 2025-07-26

**Authors:** M. Iveth Garcia, Bhavya Bhardwaj, Keri Dame, Verena Charwat, Brian A. Siemons, Ishan Goswami, Omnia A. Ismaiel, Sabyasachy Mistry, Tromondae K. Feaster, Kevin E. Healy, Alexandre J. S. Ribeiro, Ksenia Blinova

**Affiliations:** 1 Division of Applied Regulatory Science, Office of Clinical Pharmacology, Office of Translational Sciences, Center for Drug Evaluation and Research, U.S. Food and Drug Administration, Silver Spring, MD 20993, USA; martha.garcia@fda.hhs.gov (M.I.G.); bhavya.bhardwaj@fda.hhs.gov (B.B.); keri.dame@gmail.com (K.D.); omnia.ismaiel@fda.hhs.gov (O.A.I.); sabyasachy.mistry@fda.hhs.gov (S.M.); tromondae.feaster@fda.hhs.gov (T.K.F.); axribeiro3@gmail.com (A.J.S.R.); 2 Department of Bioengineering, California Institute for Quantitative Biosciences (QB3), University of California at Berkeley, Berkeley, CA 94720, USA; verena.charwat@jku.at (V.C.); briansiemons3@gmail.com (B.A.S.); ishangoswami@berkeley.edu (I.G.); kehealy@berkeley.edu (K.E.H.); 3 Department of Materials Science and Engineering, University of California at Berkeley, Berkeley, CA 94720, USA

**Keywords:** new approach methodologies (NAMs), hiPSC-CM, contractility, calcium handling, electrophysiology, cardiomyocytes, nonclinical study, complex in vitro model, microphysiological systems (MPSs)

## Abstract

New approach methodologies (NAMs), including microphysiological systems (MPSs), can recapitulate structural and functional complexities of organs. Vanoxerine was reported to induce cardiac adverse events, including torsade de points (TdP), in a Phase III clinical trial. Despite earlier nonclinical animal models and Phase I–II clinical trials, events of QT prolongation or proarrhythmia were not observed. Here, we utilized cardiac NAMs to evaluate the functional consequences of vanoxerine treatment on human cardiac excitation–contraction coupling. The cardiac MPS used in this study was a microfabricated fluidic culture platform with human-induced pluripotent stem cell-derived cardiomyocytes (hiPSC-CMs) capable of evaluating voltage, intracellular calcium handling, and contractility. Likewise, the hiPSC-CM comprehensive in vitro proarrhythmia assay (CiPA) was employed based on multielectrode array (MEA). Vanoxerine treatment delayed repolarization in a concentration-dependent manner and induced proarrhythmic events in both NAM platforms. The complex cardiac MPS displayed a frequency-dependent vanoxerine response such that EADs were eliminated at a faster pacing rate (1.5 Hz). Moreover, exposure analysis revealed a 99% vanoxerine loss in the cardiac MPS. TdP risk analysis demonstrated high to intermediate TdP risk at clinically relevant concentrations of vanoxerine and frequency-independent EAD events in the hiPSC-CM CiPA model. These findings demonstrate that nonclinical cardiac NAMs can recapitulate clinical outcomes, including detection of vanoxerine-induced delayed repolarization and proarrhythmic effects. Moreover, this work provides a foundation to evaluate the safety and efficacy of novel compounds to reduce the dependence on animal studies.

## 1. Introduction

Cardiotoxicity is one of the most common adverse drug effects and a major contributor to drug attrition in clinical trials [1,2]. Nonclinical safety and efficacy assessment of new drugs is critical to ensuring clinical success, particularly when considering the potential for cardiotoxicity. As a result, there is a growing need for more predictive, human-relevant nonclinical models to assess drug-induced cardiotoxicity. Traditional animal models often fail to accurately reflect the complexities of human cardiac biology, underscoring the importance of new approach methodologies (NAMs) in drug safety testing.

NAMs, including microphysiological systems (MPSs), represent cutting-edge technologies that mimic physiologically relevant microenvironments and enable the recreation of the structural and functional complexities of human organs [3,4,5]. These platforms provide a more accurate representation of human cardiac function and allow for the assessment of key processes in excitation–contraction (EC) coupling, such as electrophysiology, intracellular calcium handling, and contractility. Human-induced pluripotent stem cells (hiPSCs) have revolutionized cardiovascular research by enabling the differentiation of hiPSCs into cardiomyocytes (hiPSC-CMs) and providing an unlimited supply of human cardiomyocytes for in vitro evaluation [6]. Culture and analytics of hiPSC-CMs have been successfully integrated into these systems and have shown great promise for studying both cardiac disease mechanisms and drug-induced cardiac effects, making them ideal for evaluating the safety of new pharmacological agents [3,4,7,8,9].

Vanoxerine, a dopamine reuptake inhibitor, has been investigated for its potential in treating disorders such as Parkinson’s disease and atrial fibrillation (AF) [10]. However, in clinical trials, vanoxerine lacked efficacy in treating Parkinson’s disease, depression, and cocaine addiction. Vanoxerine is also a potent hERG channel blocker with the potential to be used as an oral antiarrhythmic agent. It has been found to induce multiple ion channel effects (MICEs) that offset its hERG block. The potency levels are ranked as hERG by 30% at 3 Hz (10 nM), 83% block of Cav1.2 at 1 Hz (300 nM), and 66% block of hNav1.5 at 3 Hz (300 nM) in HEK293 and guinea pig ventricular myocytes [11]. As such, vanoxerine exhibits frequency-dependent block, with potency increasing as stimulation frequency rises, particularly within the 0.3 to 3 Hz range. Studies have shown that vanoxerine can effectively terminate AF and atrial flutter (AFL), with a positive therapeutic index and with no major side effects at therapeutic concentrations [11,12]. Despite early-stage clinical trials showing favorable outcomes, Phase III trials have revealed torsades de pointes (TdP) and other adverse events, raising concerns about vanoxerine’s safety profile [13]. These findings were unexpected, given the earlier nonclinical animal models did not suggest proarrhythmic risk, and no proarrhythmic risk was observed in Phase I–II clinical trials despite vanoxerine-induced QT interval prolongation [12,14,15,16].

This study aims to leverage a complex cardiac MPS and the hiPSC-CM CiPA model to evaluate the functional consequences of vanoxerine treatment on human cardiac EC coupling. Our findings demonstrate that nonclinical cardiac NAMs can recapitulate known mechanisms of cardiotoxic drugs, including detection of vanoxerine-induced delayed repolarization and prediction of proarrhythmia potential. Through the application of these advanced NAM platforms, we seek to better understand vanoxerine’s cardiotoxicity and highlight the utility of these models for predicting drug-induced arrhythmias before clinical testing.

## 2. Materials and Methods

### 2.1. Complex Cardiac MPS Cell Source

WTC11-GCaMP6f hiPSCs were received from the Conklin Lab at the Gladstone Institutes, University of California San Francisco [17]. This line is derived from the WTC11 hiPSC line, which is commercially available from the Coriell Institute for Biomedical Research (Catalog #GM25256) and was originally reprogrammed from fibroblasts. The cell line was developed from a 30-year-old “healthy” male donor with no reported history of cardiac disease.

### 2.2. Cardiac Differentiation

WTC11-GCaMP6f hiPSCs were thawed and subsequently passaged three times in mTeSR-1 media (StemCell Technologies (Vancouver, BC, Canada), cat. no. 85870) with 10 µM ROCK inhibitor (StemCell Technologies, cat. no. 72304). Following expansion, cells were seeded for differentiation. Prior to differentiation, cell quality was assessed via flow cytometry using the pluripotency marker SSEA-3. Cardiac differentiation was initiated using small molecule modulation of the Wnt signaling pathway [6,18]. On day 0, cells at full confluency (100%) were treated with 8.5 µM CHIR99021 (LC Laboratories (Woburn, MA, USA), cat. no. C-6556) in RPMI 1640 medium containing B-27 supplement without insulin (RPMI-I, Gibco (Grand Island, NY, USA), cat. no. A1895601). CHIR99021 concentration was empirically optimized [3]. On day 2, the medium was replaced with RPMI containing IWP4 (Wnt signaling inhibitor, Stemgent (Beltsville, MD, USA), cat. no. 04-0036) and 150 μg/mL L-ascorbic acid (LAA, Fisher Scientific (Waltham, MA, USA), cat. no. AC105021000) for 48 h. From day 6 onward, cells were maintained with RPMI containing B-27 supplements with insulin (Gibco, cat. No. 17504044) until spontaneous beating was observed, typically by day 7 after differentiation. Cardiac differentiation efficiency was assessed by flow cytometry using cardiac troponin T (cTnT) as a marker. Only wells with ≥80% cTnT-positive hiPSC-CMs were used for loading into the complex cardiac MPS. Cardiac differentiation and preparation protocols are described in detail in a previously published manuscript [3].

### 2.3. Loading of the Complex Cardiac Microphysiological System

Protocols for cell singularization, dye loading, maturation media (MM) media preparation, and maintenance of the cardiac MPS were performed as previously described [3,19]. Briefly, hiPSC-CMs were singularized 7–10 days post-differentiation using collagenase type II (Worthington (Lakewood, NJ, USA), cat. no. LS004176), then resuspended in EB20 medium supplemented with ROCK inhibitor. Cells were loaded into cardiac MPS chambers at a density of 21,900 viable cells per chamber. Following loading, each device was supplied with 200 μL of EB20 medium containing 10 µM ROCK inhibitor via the inlet and incubated for 48 h. After this period, the medium was replaced with MM, and tissues were allowed to mature for 10 days prior to experimentation [3,19]. MM composition: glucose-free RPMI basal media, 0.42 mM Ca^2+^, supplemented with 2% B-27, 0.5 g/L glucose, 10 mM galactose, 2.25% BSA, 0.2 mM oleic acid, 100 μM palmitic acid, and 150 μg/mL ascorbic acid. Chamber quality was assessed before initiating experiments. This included tissue viability via live/dead staining, evaluation of intracellular calcium fluorescence expression, and observation of contractile activity. Chambers that were incompletely filled or did not meet quality criteria were excluded.

### 2.4. Pharmacological Studies

For a detailed protocol to image membrane voltage, intracellular calcium, and contractility, please refer to our previous study [3]. Briefly, voltage recordings were conducted using the synthetic dye BeRST-1 (kindly provided by Dr. Evan W. Miller, UC Berkeley) [20]. Cardiac MPS tissues were stained with 500 nM BeRST-1 voltage dye 24 h prior to pharmacological testing. On the day of the experiment, vanoxerine dihydrochloride (APExBio (Houston, TX, USA), cat. no. B3248) and sotalol hydrochloride (APExBio, cat. no. B3341) were dissolved in DMSO to prepare stock solutions of 10 mM and 1 M, respectively. Stocks were freshly prepared and serially diluted in MM containing 50 nM BeRST-1, with the final DMSO concentration adjusted to 0.1%. Before drug treatment, cardiac MPS devices were placed on a heated microscope insert P Lab-Tek 2000 (PECON (Erbach, Germany), cat. no. 2000) at 37 °C for 20 min to equilibrate. Automated fluid delivery was achieved using the Fluigent Aria system (Fluigent (Le Kremlin-Bicêtre, France), cat. no. CB-SY-AR-M-01), allowing programmable perfusion of up to 10 different solutions at a controlled flow rate of 30 μL/min. The system was primed with each drug concentration per the manufacturer’s instructions, and the outlet tubing was connected via stainless-steel connectors to the MPS inlet.

Electrical pacing was achieved by connecting alligator clips from a MyoPacer Field Stimulator (IonOptix (Westwood, MA, USA), cat. no. P0001) to the stainless-steel needles (Instech (Plymouth Meeting, PA, USA), cat. no. SC22/15) inserted into the MPS inlet and outlet. Once the setup was complete, tissues were stabilized for an additional 30 min. Baseline recordings of contractility, intracellular calcium, and membrane voltage were collected. The MPS was then perfused with escalating concentrations of each compound, with 30 min incubation at each concentration prior to data acquisition, continuing until the highest concentration was reached. Time in culture had negligible effects on the cardiac MPS parameters evaluated (Table S1).

### 2.5. Image Acquisition

Videos of the cardiac MPS were captured in both brightfield and fluorescence modes using an Axio Observer 7 inverted microscope (Carl Zeiss Microscopy (White Plains, NY, USA)) equipped with a Zeiss Colibri LED light source and an ORCA-Flash4.0 V3 Digital CMOS camera. Imaging was performed with a 20× objective at 66 frames per second (fps) at 37 °C, and data were acquired using ZEN 3.2 software (blue edition).

### 2.6. Data Analysis

Membrane voltage and intracellular calcium were analyzed using cardiac MPS version 2.1 (Simula Research Laboratory; core developer Henrik Finsberg), which is publicly available at Zenodo. Tissue motion was analyzed from brightfield videos using a MATLAB-based graphical interface with digital image correlation [21]. In this study, eleven different parameters were measured from the three optical signals that were simultaneously recorded under each condition. These included (1) action potential duration at 30% (APD30), (2) APD at 90% (APD90), (3) APD at 80% (APD80), (4) triangulation, (5) calcium transient duration at 30% (CaTD30), (6) CaTD at 90% (CaTD90), (7) calcium decay, (8) contraction displacement, (9) contraction velocity, (10) relaxation velocity, and (11) beats per min (BPM). Contraction and relaxation velocities corresponded to the maxima and minima of the average velocity at each time point [21]. Action potential triangulation was calculated as the difference between APD90 and APD30, normalized to APD90. Delta (Δ) values were calculated relative to control for each parameter. For spontaneous conditions, Fridericia’s correction was used to correct for beat rate.

### 2.7. MEA Recordings

Cryopreserved human-induced pluripotent stem cell-derived cardiomyocytes (hiPSC-CMs) (iCell Cardiomyocytes^2^ 01434, R1017, Lot Number: 107486, Fujifilm Cellular Dynamic, Inc. (Madison, WI, USA)) were thawed and plated, as previously described according to the manufacturer’s instructions [22]. iCell Cardiomyocytes^2^ were derived from a hiPSC line, which was reprogrammed from fibroblast donor tissue isolated from an apparently “healthy” normal Caucasian female aged <18 years old [23,24]. HiPSC-CM multielectrode array (MEA) (Maestro, Axion BioSystems (Atlanta, GA, USA)) recording were performed as previously described [25]. Briefly, pharmacological testing was performed on day 7 of plating cells on the MEA plates. One day prior to the experiment, hiPSC-CMs were fed with fresh maintenance media. Concentrated drug stocks were then made into 10xstocks by diluting with FluoroBrite™ DMEM media (ThermoFisher (Waltham, MA USA)). Separate compound treatment plates were prepared and equilibrated at 37 °C and 5% CO_2_. One hour before experiments, the medium was changed to 270 μL of the FluoroBrite™ DMEM media and allowed to equilibrate at 37 °C and 5% CO_2_. MEA plates were equilibrated on the Maestro system (Axion Biosystems) for 20 min before baseline recordings. Drugs were then added by pipetting 30 μL of the 10x stock solution into each well containing 270 μL of FluoroBrite™ DMEM, 1.8 mM Ca^2+^. The final concentration of DMSO did not exceed 0.1%. Post-compound data were collected 1 h after application. Field potentials in hiPSC-CMs were recorded using AxIS software version 2.4.2 (Axion Biosystems) in cardiac standard configuration (130x gain) with a sampling frequency of 12.5 kHz and a bandpass filter of 0.1–2000 Hz. A statistics compiler tool filtered the beats with spike amplitude (SA) > 0.3 mV, beat-to-beat field potential duration (FPD) consistency within 2x SD, electrode FPD consistency (10% coefficient of variation), and well FPD consistency within 2x median absolute deviation. Thirty stable beats from each recording were selected for analysis using the Axis CiPA analysis tool (version 1.2.3). Double delta (ΔΔ) FPD was calculated after correcting for compound-induced FPD with plate and timepoint vehicle control and well-based baseline FPD values [22]. TdP risk (Model 1, Dichotomous Model) was employed to predict TdP risk category as low vs. high or intermediate (Table S2) based on compound electrophysiological response as model predictors [22].

### 2.8. LC-MS/MS Analysis

LC-MS/MS methods were developed to evaluate vanoxerine and sotalol loss in the cardiac MPS due to binding to PDMS and perfusion tubing. Samples were analyzed on 1290 Infinity II UPLC (Agilent (Santa Clara, CA, USA)) and SCIEX^®^ 6500+ (SCIEX (Marlborough, MA, USA)) triple quadrupole mass spectrometer. Vanoxerine and the analogue internal standard (verapamil-d_3_) were separated on a Kinetex XB-C_18_, 2.1 × 50 mm, 1.7 μm column, using 0.1% formic acid in water (mobile phase A) and acetonitrile with 0.1% formic acid (mobile phase B). Sotalol and internal standard (sotalol-d_6_) were isolated on an Acquity RP Shield 2.1 × 50 mm, 1.7 µm column, using 10 mM ammonium formate in water (mobile phase A) and acetonitrile with 0.1% formic acid (mobile phase B). Stock solutions were prepared in DMSO and 1:1 DMSO/water (*v*/*v*) for vanoxerine and sotalol, respectively, and stored at −80 °C. Drug adsorption to the cardiac MPS was evaluated using a cell-free setup. Test solutions were perfused through empty cardiac chips for 20–30 min at 37 °C and compared to control samples that were not perfused through the MPS. Samples for LC-MS/MS analysis were collected in protein low-binding tubes and stored at −80 °C. On the day of the analysis, samples were thawed, diluted with acetonitrile, and injected into LC-MS/MS.

### 2.9. Statistical Analysis

All statistical analyses were performed using GraphPad Prism 8 software (Prism 8, GraphPad Software (Boston, MA, USA)). Differences among the groups are presented as the mean ± standard error of the mean (SEM). Differences between groups were assessed by one-way analysis of variance (ANOVA) with Tukey correction for multiple comparisons. The nonparametric Kruskal–Wallis test was used for data that were not normally distributed. Results were considered statistically significant if the *p*-value was less than 0.05.

## 3. Results

### 3.1. Vanoxerine Delays Repolarization and Induces EADs in the Complex Cardiac MPS

**Figure 1:**
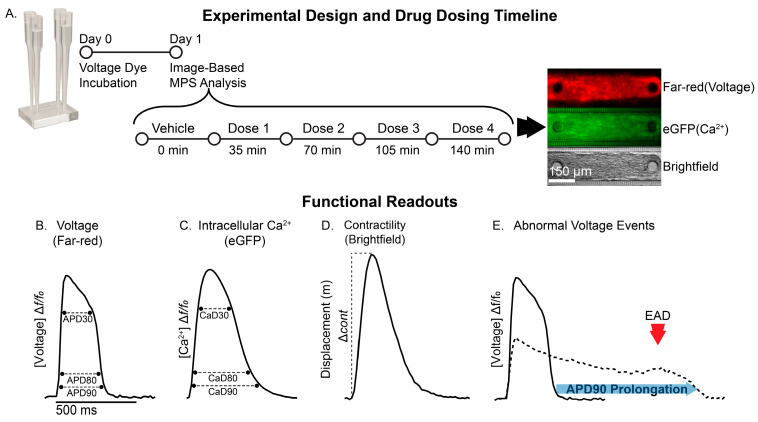
Cardiac MPS. (**A**) Schematic of the MPS experimental design for testing drug effects: Day 0, incubation with voltage-sensitive dye; Day 1, initiation of acute treatment schedule over a span of 140 min. Representative images of voltage (red), intracellular calcium (green), and brightfield (20×, scale bar 150 µm). (**B**–**D**) Representative traces of sequential measurements of (**B**) voltage-sensitive dye (far-red), (**C**) genetically encoded calcium indicator (GECI) GCaMP6f (eGFP), and (**D**) contractility waveforms, depicting key cardiac excitation–contraction coupling parameters. (**E**) Abnormal action potential characterized by APD90 prolongation (dotted lines) and EAD-like events (red arrow).

**Figure 2:**
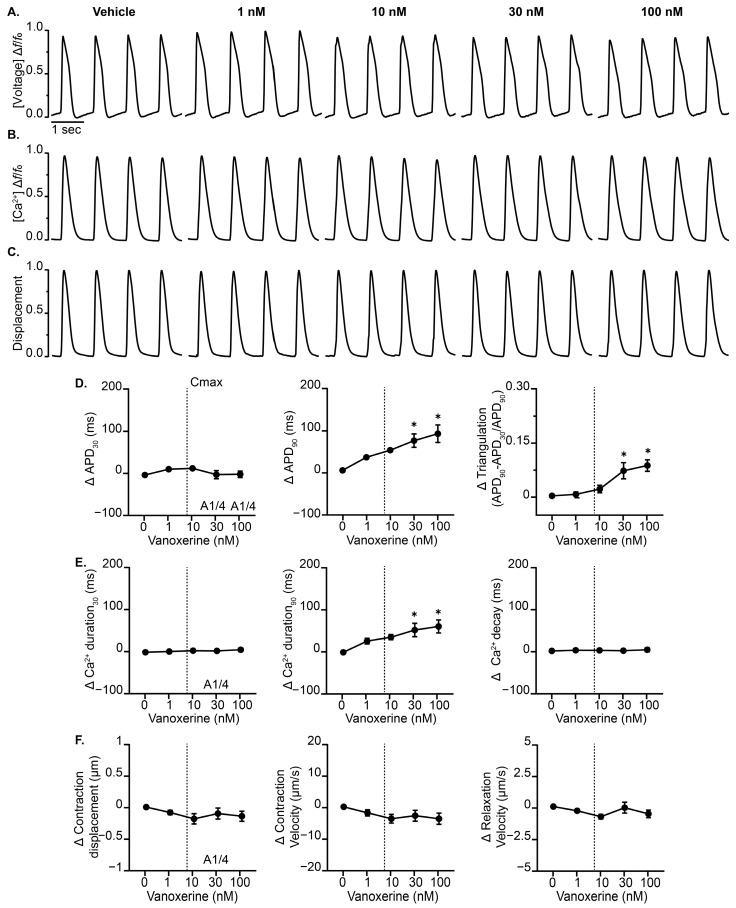
Effect of vanoxerine on cardiac MPS at 1 Hz field stimulation. Averaged waveforms of (**A**) voltage, (**B**) intracellular calcium, and (**C**) contractility recorded from cardiac MPS tissues treated with increasing concentrations of vanoxerine and paced at 1 Hz. (**D**) Summary data graphs of action potential durations (30% and 90%), as well as triangulation (APD90 − APD30) normalized to APD90. (**E**) Calcium transient durations (CaTD30 and CaTD90) and calcium decay. (**F**) Measurements of contraction displacement, contraction velocity, and relaxation velocity. A = arrhythmic events. Data are presented as mean ± SEM, *n* = 4. * *p* < 0.05 (treatment vs. vehicle). The vertical dotted line indicates the estimated unbound therapeutic Cmax of vanoxerine.

The cardiac MPS is a microfabricated fluidic platform (Figure 1A) designed to model heart tissue function. Baseline and functional characterization of the cardiac MPS used in our experiments have been reported elsewhere [3,5,9,19] and were not investigated here. First, the effects of vanoxerine on human cardiac excitation–contraction (EC) coupling readouts (Figure 1B) in vitro under spontaneous conditions were evaluated. Vanoxerine treatment significantly reduced the spontaneous beat rate of cardiac MPS relative to vehicle control (Table 1). Vanoxerine induced irregular beating morphologies, which precluded sufficient replicates for meaningful statistical analysis of APD90cF under spontaneous conditions. As such, APD80cF was evaluated to elucidate potential vanoxerine-induced effects on cardiac MPS repolarization. Vanoxerine treatment significantly delayed early (i.e., APD30cF) and late repolarization (i.e., APD80cF) in a concentration-dependent manner relative to vehicle control. Vanoxerine treatment resulted in significant triangulation of the AP morphology at 30 nM (uncorrected for nonspecific binding) with early afterdepolarization (EADs) (Table 1). Consistent with the vanoxerine-induced delayed repolarization detected by membrane voltage (VSO), vanoxerine prolonged calcium transient durations 30 and 80. Under spontaneous conditions, EAD-like events were detected as early as 10 nM by voltage, calcium, and contractility readouts (Table 1 and Table S3), consistent with increased TdP risk categorization (Table S2). To control for vanoxerine-induced effects on spontaneous beat rate, cardiac MPS were then evaluated under field stimulation (1 Hz) conditions (Figure 2). Cardiac MPS displayed a vanoxerine-induced prolongation of APD90 and AP triangularization when field stimulated at 1 Hz relative to vehicle control (Figure 2D). EAD-like events were again detected at 30 nM by voltage, calcium, and contractility readouts. Vanoxerine had negligible effects on calcium transient duration 30, whereas calcium transient duration 90 was prolonged relative to vehicle control. Vanoxerine treatment displayed negligible effects on contractile parameters relative to vehicle control (Figure 2F). These results suggest that vanoxerine-induced proarrhythmic effects can be identified in the cardiac MPS, including delayed repolarization, triangularization, and proarrhythmic events by multiple independent readouts.

### 3.2. Vanoxerine Effects Display Frequency Dependence in Cardiac MPS

**Figure 3:**
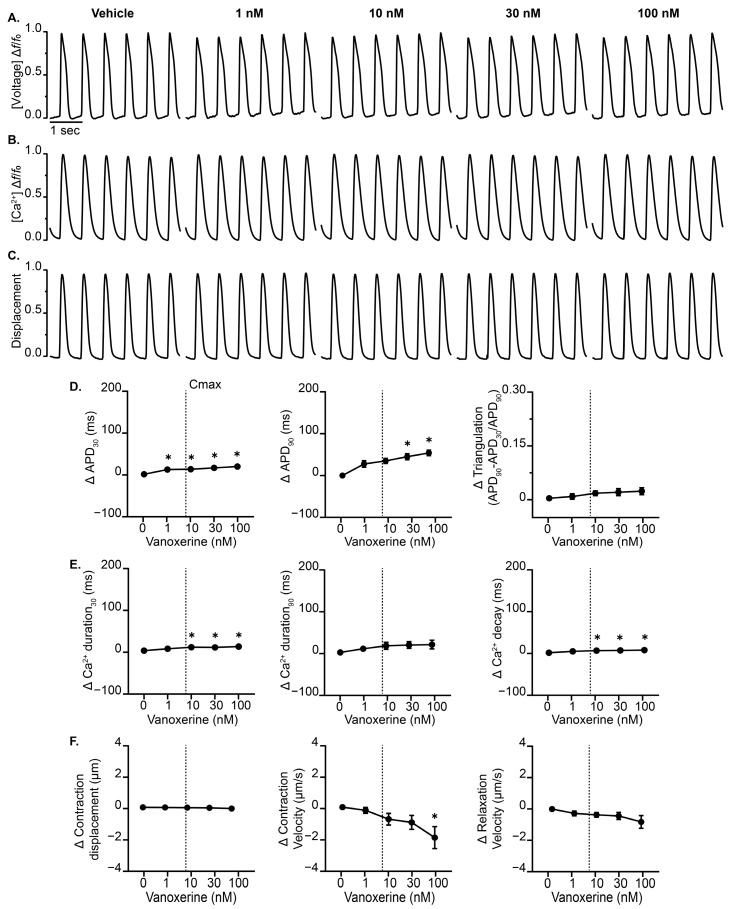
Effect of vanoxerine on cardiac MPS at 1.5 Hz field stimulation. Averaged waveforms of (**A**) voltage, (**B**) intracellular calcium, and (**C**) contractility recorded from cardiac MPS tissues treated with increasing concentrations of vanoxerine and paced at 1.5 Hz. (**D**) Summary data graphs of action potential durations (30% and 90%), as well as triangulation (APD90 − APD30/APD90). (**E**) Calcium transient durations (CaTD30 and CaTD90) and calcium decay. (**F**) Measurements of contraction displacement, contraction velocity, and relaxation velocity. Data are presented as mean ± SEM, *n* = 4. * *p* < 0.05 (treatment vs. vehicle). The vertical dotted line indicates the estimated unbound therapeutic Cmax of vanoxerine.

To determine the frequency dependence of the vanoxerine-induced EC coupling effects, we evaluated the vanoxerine treatment at a faster pacing rate (1.5 Hz). Using the same experimental setup, cardiac MPS displayed a vanoxerine-induced prolongation of APD30 and APD90 with negligible effects on AP triangulation (Figure 3). Similarly, vanoxerine prolonged calcium transient duration 30 and had a negligible effect on calcium transient duration 90 but significantly prolonged calcium decay as early as 10 nM. Vanoxerine treatment reduced contraction displacement and accelerated contraction velocity at 100 nM while having a comparable effect on relaxation velocity relative to vehicle control. EAD events were eliminated for all three cardiac EC coupling readouts when paced at 1.5 Hz. These results suggest that at faster pacing rates, vanoxerine-induced effects are mitigated, including AP triangularization and EAD-like events.

### 3.3. Effects of Cardiotoxic Drugs with Known Mechanism on the Complex Cardiac MPS

**Figure 4:**
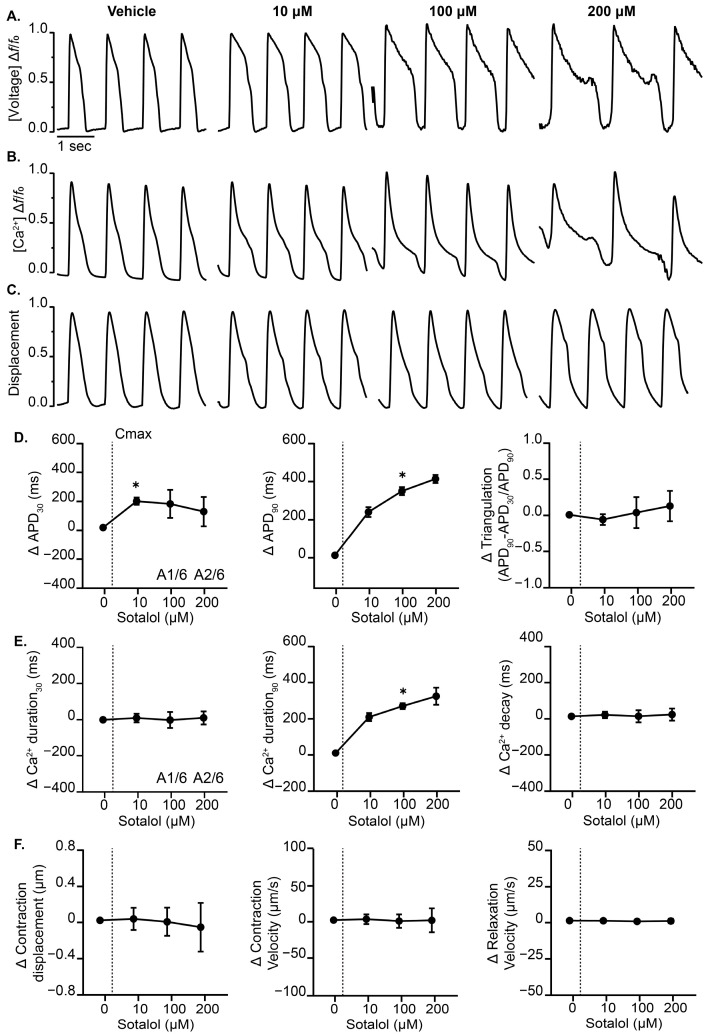
Effect of sotalol on cardiac MPS. Representative waveforms of (**A**) voltage, (**B**) intracellular calcium, and (**C**) contractility recorded from cardiac MPS tissues treated with increasing concentrations of sotalol and paced at 1 Hz. (**D**) Summary data graphs of action potential durations (30% and 90%), as well as triangulation (APD90 − APD30/APD90). (**E**) Calcium transient durations (CaTD30 and CaTD90) and calcium decay. (**F**) Measurements of contraction displacement, contraction velocity, and relaxation velocity. A = arrhythmic events. Data are presented as mean ± SEM, *n* = 6. * *p* < 0.05 (treatment vs. vehicle). The vertical dotted line indicates the estimated therapeutic Cmax of sotalol.

Next, we evaluated the utility of the complex cardiac MPS to detect known pharmacological agents. Consistent with previous reports, the high TdP risk hERG blocker sotalol (Figure 4) significantly prolonged early repolarization (APD30) and late repolarization (APD90) and resulted in robust EADs at 100 µM and 200 µM [9]. Sotalol did not affect AP triangularization. Sotalol treatment prolonged calcium transient duration 90, while all other calcium and contractility parameters were unchanged relative to vehicle control. Likewise, the high TdP risk drug dofetilide (Figure S1) resulted in EADs as early as 3 nM in all three readouts. These data suggest that the complex cardiac MPS responds predictably to hERG blockers with known cardiac effects.

### 3.4. Complex Cardiac MPS Exposure Analysis

Given that vanoxerine has been shown to have significant plasma protein binding, we next quantified the drug absorbance for vanoxerine in the complex cardiac MPS by LC-MS. Nonspecific binding resulted in significant drug loss (99%) observed for vanoxerine in the complex cardiac MPS for all concentrations tested (Table 2). For comparison, sotalol, which is thought not to have significant plasma binding, displayed negligible drug loss. These results suggest that the complex cardiac MPS and components (e.g., serum, tubing, and PDMS) may alter drug concentrations in vitro.

### 3.5. Effects of Vanoxerine on the hiPSC-CM MEA CiPA Model

**Figure 5:**
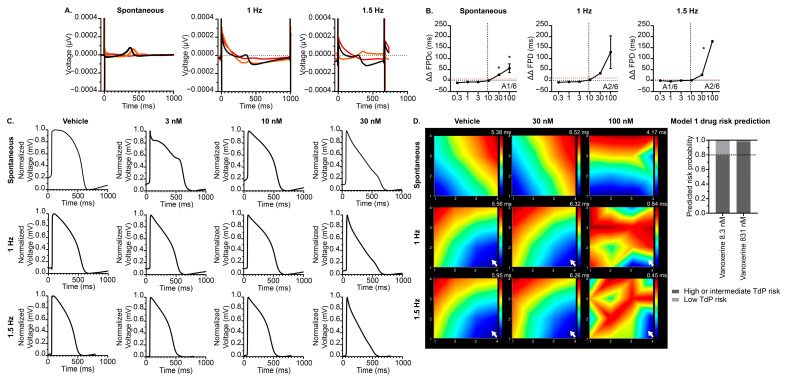
Effect of vanoxerine on hiPSC-CMs CiPA model electrophysiology. (**A**) Representative field potential recordings from vanoxerine-treated hiPSC-CMs at various frequencies (spontaneous, 1 Hz, and 1.5 Hz) for vehicle (black), vanoxerine 30 nM (orange), and vanoxerine 100 nM (red). (**B**) Summary data graphs of field potential duration (ΔΔFPD). Data are presented as mean ± SEM. *n* = 6 to 12 per condition. The vertical dotted line indicates the estimated unbound therapeutic Cmax of 8.3 nM. * *p* < 0.05 (treatment vs. vehicle), and the horizontal dotted line (red) represents the 2SD threshold. (**C**) Example local extracellular action potential (LEAP) recordings from vanoxerine-treated hiPSC-CMs at various frequencies (spontaneous, 1 Hz, and 1.5 Hz). (**D**) Representative conduction maps of the propagation delay; blue indicates the initiation and red indicates the termination. The white arrow represents pacing site (left panel) and TdP prediction risk categorization (right panel), and the horizontal dotted line represents the 0.8 threshold [22]. FPDcF = field potential duration (Fridericia correction), A = arrhythmic events.

Next, we investigated the effects of vanoxerine on a previously validated cardiac NAM under serum-free conditions to account for potential nonspecific binding. Cardiotoxic drugs with known mechanisms, including sotalol and dofetilide, have previously been investigated using this model [22] and were not investigated here. As expected, following vanoxerine treatment, hiPSC-CMs displayed significantly delayed repolarization in a concentration-dependent manner at various pacing rates relative to vehicle control (Figure 5A,B) (100 nM 1 Hz, *p* = 0.0502). There was no significant difference in the magnitude of the vanoxerine-induced prolongation at 100 nM at any frequency tested. Furthermore, we found EAD-like events as early as 1 nM at 1.5 Hz and at 100 nM for each frequency tested. Likewise, vanoxerine significantly slowed spontaneous beat rate (Table S2) relative to vehicle control. Qualitative assessment of local extracellular action potential (LEAP) morphology revealed EAD-like events at 3 nM under spontaneous conditions and triangularization at 30 nM for all conditions. Moreover, conduction maps illustrate vanoxerine-induced irregular beating patterns (Figure 5D) at 100 nM, consistent with increased TdP predicted risk categorization of vanoxerine at clinically relevant concentrations (Cmax of 8.3 nM and peak Cmax of 831 nM) (Figure 5D, Table S2). Taken together, these data demonstrate that vanoxerine treatment delays repolarization at multiple pacing rates, alters AP morphology, and induces proarrhythmic events, resulting in increased predicted TdP risk categorization in vitro.

## 4. Discussion

### 4.1. Nonclinical Prediction of Vanoxerine-Induced Proarrhythmia Risk

In this study, we used robust cardiac NAMs to quantify and detect previously uncharacterized cardiac TdP adverse effects of vanoxerine. Clinically, vanoxerine was associated with TdP and an increased risk of ventricular pro-arrhythmia in patients with structural heart disease, leading to the discontinuation of clinical trials due to safety concerns [13]. However, the cardiotoxic effects of vanoxerine treatment have not been completely defined in an in vitro model. Here, we quantified the functional effects of clinically relevant concentrations of vanoxerine in vitro using cardiac NAMs coupled with noninvasive video-based imaging and MEA platforms. We demonstrated that vanoxerine markedly delayed repolarization in a concentration-dependent manner in two independent nonclinical cardiac NAMs utilizing cells derived from two “healthy” donors. Likewise, vanoxerine induced robust EAD-like events at multiple concentrations and beat rates that were detected by multiple independent readouts (e.g., VSO, calcium, contractility, and LEAP). Moreover, EAD-like events and prolongation were more pronounced under spontaneous conditions in the complex cardiac MPS, and these features were blunted with higher pacing rates. These findings are consistent with vanoxerine’s multiple ion channel blocking properties as previously described [14], suggesting that, at low nanomolar concentrations (10 nM), vanoxerine predominantly blocks hERG channels, with lesser effect on sodium and calcium channels. This results in more prominent EAD-like events and triangularization at intermediate concentrations (30 nM) (Table 1). On the other hand, at higher vanoxerine concentrations (100 nM), EADs become less frequent, and triangularization is reduced due to inward currents beginning to be blocked (Table 1). Notably, TdP risk categorization prediction suggests high or intermediate risk liability at clinically relevant vanoxerine concentrations. These findings demonstrate cardiac NAMs have the potential to be used for safety and efficacy assessment with the advantage of minimizing the use of animal models.

Our report is the first study that systematically quantifies vanoxerine proarrhythmia (i.e., TdP) potential in multiple cardiac NAMs. The hiPSC-CM MEA CiPA model is a validated [22], high-throughput tool that enables rapid evaluation of drug-induced electrophysiological effects in 2D monolayers. Additionally, the cardiac MPS described here provides the capability to sequentially measure membrane voltage, intracellular calcium, and contractility by offering a comprehensive view of how drugs may disrupt the tightly linked processes involved in EC coupling [3,5], albeit at lower throughput. The cardiac MPS utilizes a triple parametric approach employing hiPSC-CM to model human cardiac function with enhanced physiological relevance and predictive power by capturing the full EC coupling cascade [27]. This integrated readout allows for detailed insights into how electrical signals translate into calcium flux and mechanical contraction while also enabling the detection of multiparametric toxicity and the early identification of subtle dysfunctions that may be missed by single-parameter systems. By delivering simultaneous, systems-level data, triple parametric imaging supports a predictive, mechanistically informative, and clinically relevant platform for cardiac research, drug development, and personalized medicine. The TdP risk categorization models in combination with hiPSC-CMs create a physiologically relevant platform that may fill the unmet needs of current models to predict drug-induced cardiovascular liability before clinical presentation and assist regulatory decision-making. In this study, independent application of two mechanistically distinct models allows for greater confidence in the in vitro assessment of vanoxerine’s pro-arrhythmogenic potential. This approach reflects a strategy of cross-validation based on consistent findings, rather than a combination for screening purposes. MEA and cardiac MPS interrogate complementary but distinct aspects of cardiac function, with MEA capturing extracellular electrophysiology in 2D monolayers and MPS offering tissue-level insights in a 3D context. This positions them as practical and scalable tools for early-stage drug safety assessment alongside additional complex in vitro models such as human cardiac organoids (hCOs) [28,29,30,31] and engineered heart tissues (EHT) [7,32,33]. Moreover, hCOs exhibit tissue-level relevance through their multicellular composition and 3D structure, promoting gene expression profiles mimicking adult human myocardium. Likewise, EHTs enable robust measurements of contractile force and reproducible drug responses, providing an effective platform for assessing multiple cardiac excitation–contraction readouts. Together, these advanced 3D models complement traditional monolayer systems by offering deeper insights into drug effects on cardiac structure and function.

### 4.2. Comparison of Nonclinical and Clinical Vanoxerine Studies

Previous nonclinical hiPSC-CM studies of vanoxerine are challenging to correlate with each other as they apply a variety of experimental conditions and protocols. However, the unifying result is that vanoxerine treatment delays repolarization. For example, a previous study utilizing 2D monolayer and quasi-3D isotropic and anisotropic constructs of hiPSC-CMs demonstrated robust prolongation but no arrhythmia based on optical voltage and calcium readouts [34]. Likewise, a study employing isolated single-cell hiPSC-CMs reported prolongation and multiple cardiac ion channel inhibition based on patch clamp experiments and no proarrhythmia markers. Of note, an EAD occurrence in at least one cell was described as an outlier event [14]; however, given recent TdP adverse events, this is likely notable. Utilizing the hiPSC-CM MEA CiPA model, researchers have demonstrated vanoxerine-induced prolongation and arrhythmia that are consistent with our findings [35]. Importantly, the effects were delayed relative to other hERG blockers, occurring after 3 h of vanoxerine treatment. In animal in vivo studies (i.e., dogs), vanoxerine treatment has been shown to terminate atrial fibrillation (AF) and, at high doses (7.5 nM), delay repolarization and decrease the heart rate [16,36]. Clinically, in the Phase II trial, vanoxerine has been associated with prolonged QTc interval (outside the estimated therapeutic range), albeit without arrhythmic events [12]. On the other hand, in a Phase III clinical study in AF patients with structural damage, TdP risk was identified following vanoxerine treatment [13]. Here, utilizing in vitro cardiac NAMs, we demonstrated consistent and reproducible vanoxerine-induced prolongation and EADs at clinically relevant concentrations in two independent “healthy” hiPSC-CM models. Moreover, our findings suggest that vanoxerine liability is independent of cardiac structural damage in cardiac NAMs in vitro. The latter result may be important for patient population selection. Taken together, cardiac NAMs have the potential to be used for safety and efficacy assessment at the nonclinical stage of the drug development process with the advantage of minimizing the use of animal models, consistent with the FDA road map to phase out animal drug testing [37].

### 4.3. Study Limitations

Our study has several limitations. For example, the hiPSC-CMs used in the study represent apparently “healthy” backgrounds, whereas vanoxerine has been investigated for several disease states, including atrial fibrillation. As such, the model can be extended to diseased backgrounds and patient-specific hiPSC-CMs. To address the key maturation limitations of standard 2D hiPSC-CMs, we leveraged a complex 3D cardiac MPS that incorporates a commercial hiPSC line differentiated simultaneously into multiple cardiac cell types including cardiomyocytes, myofibroblasts, fibroblasts, endothelial cells, and smooth muscle cells [3], along with enhanced fatty acid maturation medium and dynamic fluidic flow. The hiPSC-CM CiPA experimental design is plate-based, utilizing commercial hiPSC-CMs coupled with static commercial MEA or VSO platforms that do not provide flow or removal of metabolic waste products. As such, we cannot exclude the impact of the complex cardiac MPS experimental configuration and conditions on the vanoxerine-induced response. We only evaluated vanoxerine acutely (e.g., 35 min per concentration); more chronic timepoints may be of interest (i.e., days to weeks), but this was out of the scope of the current study. The two NAMs were not evaluated in parallel under identical experimental conditions, and as such, we cannot directly compare their results, limiting data correlations. The cardiac MPS enables multiple independent cardiac readouts (sequential) in one assay. While not evaluated here, hiPSC-CM MEA platforms, including the one used in this study, are often capable of multiplexing with additional readouts, such as contractility (e.g., impedance) or calcium probes, to enhance mechanistic elucidation. Finally, when utilizing in vitro cardiac NAMs, important factors to consider are nonspecific binding of compounds to highly hydrophobic materials, which can disrupt actual drug concentrations, data interpretations, and clinical relevance. As such, future experimental design should include platform and well exposure analysis to determine the amount of nonspecific binding and, when necessary, evaluate drug accumulation in the cardiomyocyte tissue or monolayer, which may not be measured in the effluent.

## 5. Conclusions

This study provides a framework for the nonclinical evaluation of compound effects on human cardiac EC coupling parameters to support safety and efficacy assessment. Here, we demonstrate several important findings: (1) vanoxerine treatment delayed repolarization in a concentration-dependent manner; (2) cardiac NAMs could predict vanoxerine-induced TdP proarrhythmic risk categorization in vitro; (3) two independent “healthy” cardiac NAMs responded appropriately to vanoxerine; (4) multiple independent cardiac EC coupling readouts could detect vanoxerine-induced EADs in the complex cardiac MPS; (5) the cardiac MPS detected EADs as early as 0.1 nM (corrected for nonspecific binding) (Table 2), suggesting enhanced EAD detection; and (6) in cardiac NAMs, vanoxerine-induced EADs developed independently of cardiac structural damage. These findings may also be useful for evaluating other late-stage compounds or therapeutic modalities whose cardiotoxic potential may have been missed in early clinical development. Taken together, the current study demonstrates that nonclinical cardiac NAMs can recapitulate clinical outcomes and predict vanoxerine-induced delayed repolarization and TdP risk categorization in vitro.

## Figures and Tables

**Table 1:** Effect of vanoxerine on complex cardiac MPS EC coupling properties under spontaneous conditions.

Parameter	1 nM	10 nM	30 nM	100 nM
Voltage				
Δ APD30cF (ms)	30.1 ± 2.9	54.7 ± 14.7 *	57.8 ± 21.7 *	60.92 ± 17.9 *
Δ APD80cF (ms)	117.0 ± 17	360.4 ± 101.4 *	425.5 ± 93.8 *	344.8 ± 66 *
Δ Triangulation	0.04 ± 0.01	0.1 ± 0.05	0.2 ± 0.05 *	0.1 ± 0.04
Observed EADs	0/4	2/4	2/4	1/4
ΔBPM	−2.9 ± 2.3	−10.6 ± 3.7 *	−7.8 ± 2.4	−11.7 ± 3.5 *
Intracellular Calcium				
Δ CaTD30cF (ms)	16.1 ± 5.8	32.3 ± 14.6	50.2 ± 32.9 *	41.2 ± 21.5 *
Δ CaTD80cF (ms)	92.5 ± 14.2	286.0 ± 106.5 *	332.5 ± 93.8 *	281.8 ± 78.9 *
Δ Ca decay (ms)	4.8 ± 1.8	1.51 ± 4.8	5.9 ± 6.9	7.9 ± 7
Observed EADs	0/4	2/4	2/4	2/4
ΔBPM	−5.1 ± 1.7	−9.5 ± 3.2	−12.1 ± 4.8 *	−8.5 ± 3.4
Contractility				
Δ Contraction displacement (µm)	−03 ± 0.03	0.06 ± 0.04	−0.09 ± 0.06	−0.1 ± 0.09
Δ Contraction velocity (µm/s)	−1.37 ± 0.7	−2.06 ± 1.1	−2.81 ± 1.4	−3.57 ± 1.9
Δ Relaxation velocity (µm/s)	−0.4 ± 0.1	−0.65 ± 0.2	−0.73 ± 0.2	−0.71 ± 0.3
Observed EADs	0/4	1/4	2/4	1/4
ΔBPM	−5.8 ± 2.2	−9.9 ± 4.1	−12.3 ± 3.9	−12.6 ± 3.9 *
N	4	4	4	4

* *p* < 0.05.

**Table 2:** Complex cardiac MPS exposure analysis.

Compound	Cmax	LogP Value (Predicted)	% Drug Loss
Vanoxerine	831 nM	4.9 and 6.24	99%
Sotalol	2.7 µM	0.85 and −0.4	0%

LogP, Cmax [14,26].

## Data Availability

The raw data supporting the conclusions of this article will be made available by the authors on request.

## Supplementary Materials

The following supporting information can be downloaded at https://www.mdpi.com/article/10.3390/jcdd12080285/s1, Figure S1. Effect of dofetilide on complex cardiac MPS; Table S1. Effect of vehicle control perfusion on EC coupling parameters in cardiac MPS under 1 Hz pacing; Table S2. Vanoxerine TdP proarrhythmic risk categorization in cardiac NAMs; Table S3. Raw effects of vanoxerine on cardiac MPS EC coupling parameters under spontaneous conditions (no baseline normalization); Table S4. Effect of vanoxerine on hiPSC-CM MEA electrophysiological properties; Table S5. Effect of vanoxerine on 2D hiPSC-CM LEAP properties.

## Abbreviations

The following abbreviations are used in this manuscript:

NAMs	New approach methodologies
MPS	Microphysiological system
TdP	Torsade de points
VSO	Voltage-sensing optical
CiPA	Comprehensive in vitro proarrhythmia assay
MEA	Multielectrode array
EC	Excitation–contraction
hiPSC-CM	Human-induced pluripotent stem cell-derived cardiomyocyte
AF	Atrial fibrillation
MICEs	Multiple ion channel effects
AFL	Atrial flutter
MM	Maturation media
BeRST	Berkeley rhodamine-based sensor of transmembrane potential
hERG	Human ether-a-go-go-related gene
Cav1.2	L-type calcium channel
hNav1.5	Human Nav1.5 sodium channel
LAA	L-ascorbic acid
cTnT	Cardiac troponin T
APD	Action potential duration
CaTD	Calcium transient duration
BPM	Beats per minute
FPD	Field potential duration
EAD	Early after depolarization
LEAP	Local extracellular action potential
Cmax	Maximum serum concentration
