# Supplementary material for: Nonclinical Human Cardiac New Approach Methodologies (NAMs) Predict Vanoxerine-Induced Proarrhythmic Potential
Source: J Cardiovasc Dev Dis. 2025 Jul 26;12(8):285. doi: 10.3390/jcdd12080285 (PMC12387051; doi:10.3390/jcdd12080285)
Supplement: Supplementary file 1 [file jcdd-12-00285-s001.zip › jcdd-3676509-supplementary.pdf]

The most recent email mentioned referring to previous correspondence, but I have not received any earlier messages regarding the timeline. I want to ensure we are aligned and on schedule.

Supplementary Information

Accompanying the protocol entitled

Nonclinical human cardiac new approach methodologies (NAMs) predict vanoxerine-induced proarrhythmic potential

M. Iveth Garcia [1], Bhavya Bhardwaj [1], Keri Dame [1], Verena Charwat [2], Brian A. Siemons [2], Ishan Goswami [2], Omnia A. Ismaiel [1], Sabyasachy Mistry [1], Tromondae K. Feaster [1], Kevin E. Healy [2,3], Alexandre J. S. Ribeiro [1], Ksenia Blinova[1*]

M. Iveth Garcia[1] (M.I.G.) – martha.garcia@fda.hhs.gov
Bhavya Bhardwaj [1] (B.B.) – bhavya.bhardwaj@fda.hhs.gov
Keri Dame [1] (K.D.) – keri.dame@gmail.com
Verena Charwat [2] (V.C.) – verena.charwat@jku.at
Brian A. Siemons [2] (B.A.S.) – briansiemons3@gmail.com
Ishan Goswami [2] (I.G.) – ishangoswami@berkeley.edu
Omnia A. Ismaiel [1] (O.I.) – omnia.ismaiel@fda.hhs.gov
Sabyasachy Mistry [1] (S.M.) – sabyasachy.mistry@fda.hhs.gov
Tromondae K. Feaster [1] (T.K.F.) – tromondae.feaster@fda.hhs.gov
Kevin Healy [2,3] (K.E.H.) – kehealy@berkeley.edu
Alexandre J.S. Ribeiro [1] (A.J.S.R) – axribeiro3@gmail.com
Ksenia Blinova [1] (K.B.) – ksenia.blinova@fda.hhs.gov

[1]Division of Applied Regulatory Science, Office of Clinical Pharmacology, Office of Translational Sciences, Center for Drug Evaluation and Research, U.S. Food and Drug Administration, Silver Spring, MD, United States.
[2] Department of Bioengineering and California Institute for Quantitative Biosciences (QB3), University of California at Berkeley, Berkeley, CA, USA.
[3] Department of Materials Science and Engineering, University of California at Berkeley, Berkeley, CA, USA.

* Correspondence:
Ksenia Blinova, ksenia.blinova@fda.hhs.gov

U.S. Food and Drug Administration
10903 New Hampshire Avenue
Silver Spring, MD 20993

Contents

Section 1. Supplementary Figures

1.1. Supplementary Figure S1 4

Section 2. Supplementary Tables

2.1. Supplementary Table S1 7

2.2. Supplementary Table S2 8

2.3. Supplementary Table S3 9

2.4. Supplementary Table S4 10

2.5. Supplementary Table S5 11

Section 3. Supplementary Information References 12

<u>Section 1. Supplementary Figures</u>

1.1. Supplementary Figure S1 .... **4**

1.1. Supplementary Figure S1

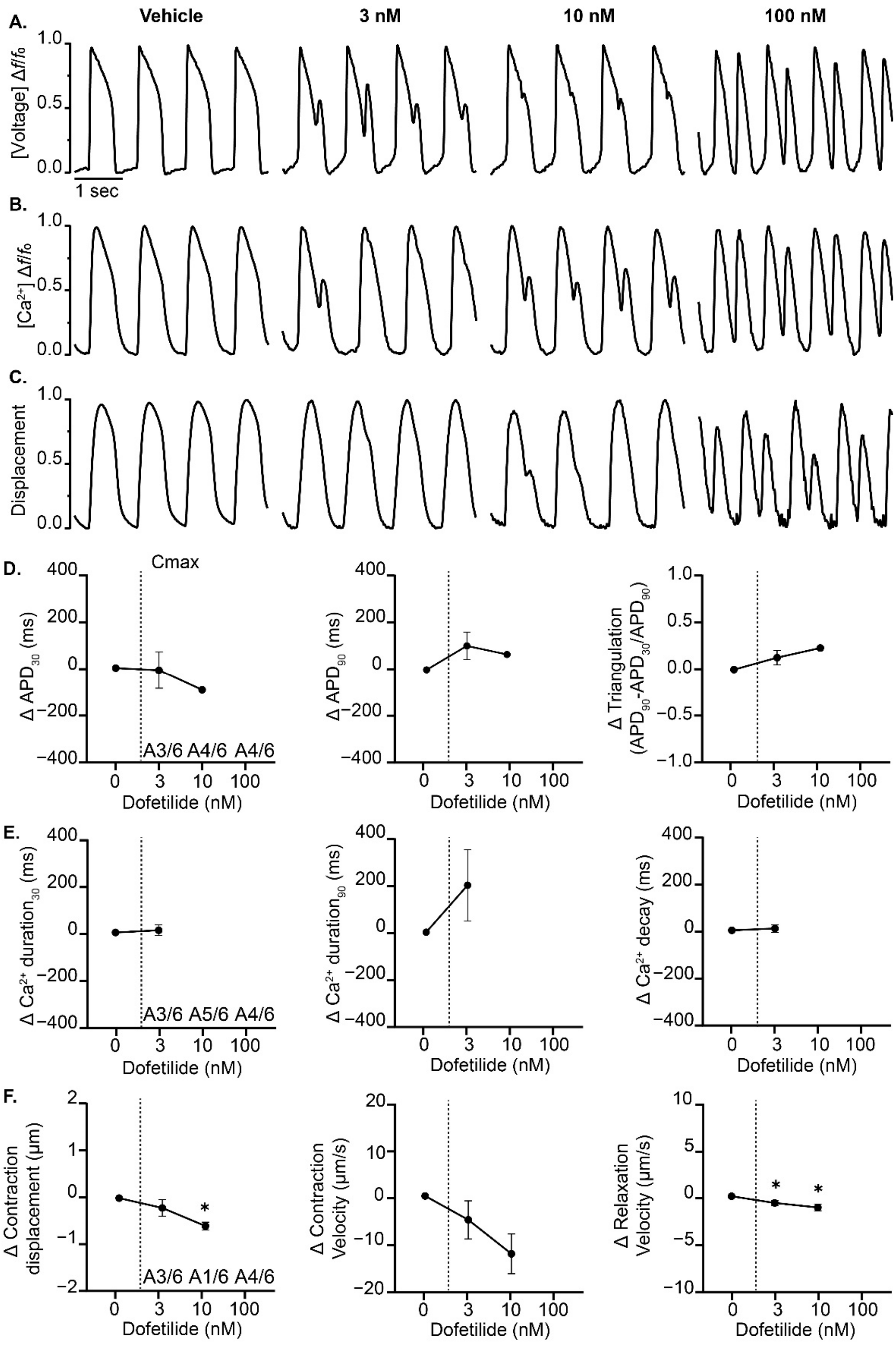

Figure S1. Effect of dofetilide on complex cardiac MPS. Representative waveforms of (A) voltage, (B) intracellular calcium, and (C) contractility recorded from cardiac MPS tissues treated with increasing concentrations of dofetilide and paced at 1Hz. (D) Summary data graphs of voltage APD30 and APD90 and triangulation show no significant changes across concentrations. However, at 10 nM Dofetilide, tissues frequently failed to respond to pacing (i.e., capture), exhibiting prolonged APDs, EADs (Type A and Type C). (E) Intracellular calcium transient duration at 30% and 90% recovery, as well as the Ca^{2+} decay, also show no significant changes. Comparable to voltage responses, pacing failure occurred at 10 nM due to prolonged calcium transients or EAD-like features. (F) Contractility analysis reveals a decrease in relaxation velocity at 3 nM Dofetilide. At 10 nM, tissues again failed to respond to pacing, consistent with findings from voltage and calcium recordings. Tissues that did not respond to electrical pacing at 10 and 100 nM were excluded from waveform analyses. Data are presented as mean ± SEM, $n = 6$. $*p < 0.05$ (Treatment vs. Vehicle).

Section 2. Supplementary Tables

2.1. Supplementary Table S1 7

2.2. Supplementary Table S2 8

2.3. Supplementary Table S3 9

2.3. Supplementary Table S4 10

2.3. Supplementary Table S5 11

2.1. Supplementary Table S1

Table S1. Effect of vehicle control perfusion on EC coupling parameters in cardiac MPS under 1 Hz pacing.

Parameter	Vehicle Timepoint 1	Vehicle Timepoint 2	Vehicle Timepoint 3	Vehicle Timepoint 4
Voltage				
Δ APD30 (ms)	5.8 ± 18.2	16.2 ± 21.4	9.1 ± 36	19.6 ± 26.6
Δ APD80 (ms)	9.1 ± 18.5	30.6 ± 15.4	24.2 ± 30.9	49.21 ± 12.1
Δ APD90 (ms)	24.8 ± 17.8	46.7 ± 14.0	52.6 ± 34.1	71.4 ± 8.6
Δ Triangulation	0.01 ± 0.03	0.03 ± 0.03	0.05 ± 0.06	0.1 ± 0.05
Observed EADs	0/3	0/3	0/3	0/3
ΔBPM	0.06 ± 0.07	0.18 ± 0.3	0.13 ± 0.2	0.04 ± 0.1
Intracellular Calcium				
Δ CaTD30 (ms)	-8.8 ± 10.4	-10.7 ± 12.5	-22.3 ± 18.4	-5.8 ± 20.1
Δ CaTD80 (ms)	-1.5 ± 19.4	8 ± 22.1	0.09 ± 35.2	13.9 ± 16.1
Δ Rate of decay (ms)	-5.04 ± 6.7	-7.3 ± 10.3	-20.1 ± 13.7	-7.3 ± 18.8
Observed EADs	0/3	0/3	0/3	0/3
ΔBPM	0.05 ± 0.1	0.08 ± 0.04	0.06 ± 0.1	0 ± 0.1
Contractility				
Δ Contraction Displacement (μm)	-0.05 ± 0.08	-0.09 ± 0.1	-0.08 ± 0.08	-0.02 ± 0.05
Δ Contraction Velocity (μm/s)	0.1 ± 0.4	-0.3 ± 0.4	-0.3 ± 0.3	-0.1 ± 0.3
Δ Relaxation Velocity (μm/s)	-0.1 ± 0.4	-0.3 ± 0.4	-0.2 ± 0.4	-0.02 ± 0.3
Observed EADs	0/3	0/3	0/3	0/3
ΔBPM	-1.48 ± 1.7	0.76 ± 0.6	1.06 ± 0.4	0.16 ± 0.5

*$p < 0.05$.

2.2. Supplementary Table S2

Table S2. Vanoxerine TdP Proarrhythmic Risk Categorization in Cardiac NAMs [1].

Parameter	Complex Cardiac MPS		hiPSC-CM MPS	
Cmax (nM)	8.3	831	8.3	831
Predictor 1, Drug-induced arrhythmia at any concentration	1, Type A	1, Type A	1, Type A	1, Type A
Predictor 4 (ms), Maximum repolarization at any concentration	425.5[1]	425.5[1]	56.4	56.4
Predictor 7 (ms), drug-induced repolarization change at Cmax	299.7[1]	373.1[1]	3.5	110.5
Cell Type	0	0	0	0
Probability of high or intermediate TdP risk	0.99997845	0.999996074	0.79	0.98
Probability of low TdP risk	2.15497E-05	3.92553E-06	0.21	0.021

[1] =Δ APD80cF.

2.3. Supplementary Table S3

Table S3. Raw Effects of Vanoxerine on Cardiac MPS EC Coupling Parameters Under Spontaneous Conditions (No Baseline Normalization).

Parameter	Vehicle	1 nM	10 nM	30 nM	100 nM
Voltage					
APD30cF (ms)	256.2 ± 35.1	286.3 ± 37.8	311.0 ± 44.5	314.1 ± 40.8	317.2 ± 36.9
APD80cF (ms)	522.0 ± 61.4	639.1 ± 74.9	882.4 ± 170.2	947.5 ± 142.8	866.9 ± 120.4
Triangulation	0.5 ± 0.04	0.6 ± 0.03	0.6 ± 0.05	0.7 ± 0.04	0.6 ± 0.02
Observed EADs	0/4	0/4	2/4	2/4	1/4
BPM	38.6 ± 3.9	35.7 ± 4.7	28 ± 5.1	31 ± 3.9	27 ± 3.8
Intracellular Calcium					
CaTD30cF (ms)	212.2 ± 19.6	228.3 ± 23.4	244.5 ± 29.4	262.4 ± 44.3	253.4 ± 33.9
CaTD80cF (ms)	474.1 ± 46.6	566.6 ± 60.3	760.1 ± 141	806.6 ± 121.6	755.9 ± 117.1
Rate of decay (ms)	108.3 ± 6.9	113.1 ± 7.9	109.8 ± 9.4	114.2 ± 12.1	116.2 ± 11.7
Observed EADs	0/4	0/4	2/4	2/4	2/4
BPM	40.2 ± 4.5	34 ± 4.4	30.1 ± 5	28 ± 5.6	31.7 ± 5.2
Contractility					
Contraction Displacement (μm)	0.8 ± 0.2	0.7 ± 0.2	0.7 ± 0.2	0.7 ± 0.2	0.7 ± 0.2
Contraction Velocity (μm/s)	14.8 ± 5.5	13.5 ± 5	12.7 ± 4.7	11.9 ± 4.3	11.2 ± 4
Relaxation Velocity (μm/s)	3.4 ± 0.9	3.0 ± 1	2.8 ± 0.8	2.7 ± 0.8	2.7 ± 0.8
Observed EADs	0/4	0/4	1/4	2/4	1/4
BPM	39.5 ± 4.2	33.7 ± 4.3	29.6 ± 5.7	27 ± 5.5	26.9 ± 4.9
N	4	4	4	4	4

* $p < 0.05$.

2.4. Supplementary Table S4

Table S4. Effect of vanoxerine on hiPSC-CM MEA electrophysiological properties.

Parameter	0.3 nM	1 nM	3 nM	10 nM	30 nM	100 nM
Spontaneous						
ΔΔ Sodium Spike Amplitude (mV)	0.8 ± 0.2	0.2 ± 0.5	0.3 ± 0.6	1.3 ± 1.0	0.1 ± 1.1	-4.4 ± 1.9*
ΔΔ Sodium Spike Slope (V/s)	-2.0 ± 0.5	-0.5± 1.4	-0.6 ± 1.4	-2.9 ± 2.4	-0.1 ± 2.6	11.0 ± 4.6*
ΔΔ Conduction Velocity (mm/ms)	0.02 ± 0.03	-0.01 ± 0.01	-0.01 ± 0.04	-0.02 ± 0.03	0.05 ± 0.06	0 .01± 0.1
ΔΔ BPM	-0.8 ± 1.6	1.3 ± 0.6	1.0 ± 0.5	-0.8 ± 1.0	-3.6 ± 2.2	-10.0 ± 4.6*
N	6	6	6	6	6	5
1 Hz						
ΔΔ Sodium Spike Amplitude (mV)	1.0 ± 0.2	0.4 ± 0.2	0.7 ± 0.3*	0.6 ± 0.2	1.1 ± 0.7*	0.4 ± 0.7
ΔΔ Sodium Spike Slope (V/s)	-3.0 ± 0.6*	-1.0 ± 0.4	-1.7 ± 0.6*	-1.4 ± 0.5	-2.8 ± 1.8*	0.005 ± 2.0
ΔΔ Conduction Velocity (mm/ms)	0.01 ± 0.002	0 ± 0	0.002 ± 0.002	0 ± 0.003	-0.003 ± 0.004	-0.03 ± 0.01
ΔΔ BPM	0 ± 0	0 ± 0	0 ± 0	0 ± 0	0 ± 0	0 ± 0
N	6	6	6	6	6	3
1.5 Hz						
ΔΔ Sodium Spike Amplitude (mV)	1.0 ± 0.2*	0.4 ± 0.1	0.9 ± 0.2*	0.7 ± 0.2	-0.2 ± 0.7	-11.0
ΔΔ Sodium Spike Slope (V/s)	-2.3 ± 0.6*	-0.9 ± 0.4	-2.2 ± 0.4*	-1.6 ± 0.6	-0.3 ± 1.9	29.1
ΔΔ Conduction Velocity (mm/ms)	0.003 ± 0.002	-0.004 ± 0.002	-0.02 ± 0.03	-17.2 ± 24.0	-0.01 ± 0.003*	-54.0
ΔΔ BPM	0 ± 0	0 ± 0	0 ± 0.002	0 ± 0	0 ± 0	0
N	12 6	5	6	6	6	1

*$p < 0.05$.

2.5. Supplementary Table S5

Table S5. Effect of vanoxerine on 2D hiPSC-CM LEAP properties.

Parameter	Vehicle	3 nM	10 nM	30 nM
Spontaneous				
APD30 (ms)	332.8	307.2	256.8	140.8
APD50 (ms)	384.8	371.2	345.6	257.6
APD90 (ms)	439.2	440	425.6	476
APD30cF (ms)	298.5	273.0	242.3	124.1
APD50cF (ms)	345.1	329.9	326.1	227.0
APD90cF (ms)	394.0	391.0	401.6	419.5
N	1	1	1	1
1.0 Hz				
APD30 (ms)	244.0 ± 1.2	220.8 ± 29.6	227.2	98.4
APD50 (ms)	327.2 ± 0.8	311.2 ± 21.6	320.8	197.6
APD90 (ms)	407.2 ± 5.6	396.8 ± 12.8	411.2	424
N	2	2	1	1
1.5 Hz				
APD30 (ms)	212.0 ± 22.0	188.8	193.6	88.8
APD50 (ms)	291.0 ± 24.4	262.4	268	172
APD90 (ms)	394.0 ± 49.0	342.4	350.4	355.2
N	2	1	1	1

Section 3. Supplementary Information References

1. Blinova, K., et al., *International Multisite Study of Human-Induced Pluripotent Stem Cell-Derived Cardiomyocytes for Drug Proarrhythmic Potential Assessment.* Cell Rep, 2018. **24**(13): p. 3582-3592.
